# Delayed-Release Dimethyl Fumarate Safety and Efficacy in Pediatric Patients With Relapsing-Remitting Multiple Sclerosis

**DOI:** 10.3389/fneur.2020.606418

**Published:** 2021-01-04

**Authors:** Raed Alroughani, Peter Huppke, Maria Mazurkiewicz-Beldzinska, Astrid Blaschek, Martin Valis, Gregory Aaen, Joe Pultz, Xiaomei Peng, Vanessa Beynon

**Affiliations:** ^1^Dasman Diabetes Institute, Dasman, Kuwait and Amiri Hospital, Sharq, Kuwait; ^2^Department of Pediatrics and Pediatric Neurology, University Medical Center Göttingen, Göttingen, Germany; ^3^Klinika Neurologii Rozwojowej, Uniwersyteckie Centrum Kliniczne, Gdansk, Poland; ^4^Department of Pediatric Neurology and Developmental Medicine, Hauner Children's Hospital, University of Munich, Munich, Germany; ^5^Neurologicka klinika, Fakultni nemocnice Hradec Kralove, Hradec Kralove, Czechia; ^6^Loma Linda University Children's Health, Loma Linda, CA, United States; ^7^Biogen, Cambridge, MA, United States

**Keywords:** relapsing-remitting multiple sclerosis, dimethyl fumarate, safety, efficacy, pediatric, pharmacokinetics

## Abstract

**Background:** Pediatric multiple sclerosis (MS) is rare: only 1.5–5% of MS cases are diagnosed before 18 years of age, and data on disease-modifying therapies (DMTs) for pediatric MS are limited. The CONNECTED study assessed the long-term safety and efficacy of treatment with delayed-release dimethyl fumarate (DMF), an oral MS DMT, in pediatric patients with MS.

**Methods:** CONNECTED is the 96-week extension to FOCUS, a 24-week phase 2 study of patients aged 13–17 years; participants received DMF 240 mg twice daily. Endpoints included (primary) incidence of adverse events (AEs), serious AEs, and DMF discontinuations due to an AE, and (secondary) T2 hyperintense lesion incidence by magnetic resonance imaging and annualized relapse rate (ARR).

**Results:** Twenty participants [median (range) age, 17 (14–18) years; 65% female] who completed FOCUS enrolled into CONNECTED; 17 (85%) completed CONNECTED. Eighteen participants (90%) experienced AEs: the most frequent was flushing (25%). None experienced infections or fever related to low lymphocyte counts. Three participants experienced four serious AEs; none led to DMF discontinuation. Twelve of 17 participants (71%) had no new/newly enlarged T2 lesions from weeks 16–24, two (12%) had one, and one each (6%) had two, three, or five or more lesions [median (range), 0 (0–6)]. Over the full 120-week treatment period, ARR was 0.2, an 84.5% relative reduction (*n* = 20; 95% confidence interval: 66.8–92.8; *p* < 0.0001) vs. the year before DMF initiation.

**Conclusions:** The long-term safety and efficacy observed in CONNECTED was consistent with adults, suggesting pediatric and adolescent patients with MS might benefit from DMF treatment.

## Introduction

Pediatric multiple sclerosis (MS) is a rare disease, with only 1.5–5% of all MS cases diagnosed before 18 years of age (1–9). Up to 98% of pediatric patients with MS present with relapsing-remitting MS (RRMS), compared with 84% of adults (1). Pediatric patients tend to have a higher frequency of relapses (10, 11) and are more likely to be hospitalized for treatment, highlighting the importance of preventing relapses through effective disease-modifying therapies (DMT) (12).

At present, starting treatment early in the disease course is recommended (13, 14), but adequate long-term safety and efficacy data on DMTs for the pediatric population are limited, resulting in a shortage of approved MS-specific treatment options in this patient population (15). The most commonly used agents in pediatric MS have been assessed almost exclusively in observational studies; therefore, there exists, a significant unmet need for studies assessing additional MS treatment options in this age group (16–18). The European Medicines Agency has granted limited approval for the use of interferon beta (IFN-β) and glatiramer acetate in patients ≥12 years of age (19). Safety data for patients ≥2 years of age is included in the European label for IFN-β-1a subcutaneous (Rebif) (18, 19). An observational study of natalizumab showed safety and efficacy in children was similar to the adult population (20). Rituximab is not approved by the US Food and Drug Administration for the treatment of patients with MS, but has been studied in small trials of pediatric patients, showing it to be safe and effective (21). Fingolimod is the only MS therapy currently approved by both the US Food and Drug Administration and the European Medicines Agency for use in pediatric patients 10–17 years of age (22) on the basis of the positive outcome of a randomized controlled trial (23).

Delayed-release dimethyl fumarate (DMF), also known as gastro-resistant DMF, has demonstrated a favorable benefit-risk profile in adults with relapsing-remitting MS in randomized, double-blind, placebo-controlled phase 3 studies (DEFINE and CONFIRM) (24–26) and a long-term extension study (ENDORSE) (27). As of June 30, 2020, >475,000 patients have been treated with DMF worldwide, representing >950,000 patient-years of exposure (28). Of these, 6,335 patients (14,241 patient-years) were treated in clinical trials (28). There have been no notable differences in the expression (29) and activity of esterases important for DMF metabolism in juveniles (12–18 years of age) vs. adults, suggesting the DMF dose approved for adults is suitable for juveniles.

There are few published articles available on the use of DMF to treat children or adolescents with MS that include safety, efficacy, and tolerability data (30). CONNECTED is the 96-week extension study of FOCUS (ClinicalTrials.gov NCT02410200), a 24-week, multicenter, phase 2 study that enrolled patients 13–17 years of age. The FOCUS study showed safety, tolerability, and pharmacokinetic profiles of DMF in pediatric patients with relapsing-remitting MS were consistent with those observed in adults (31).

## Methods

### Study Design and Participants

**Figure 1:**
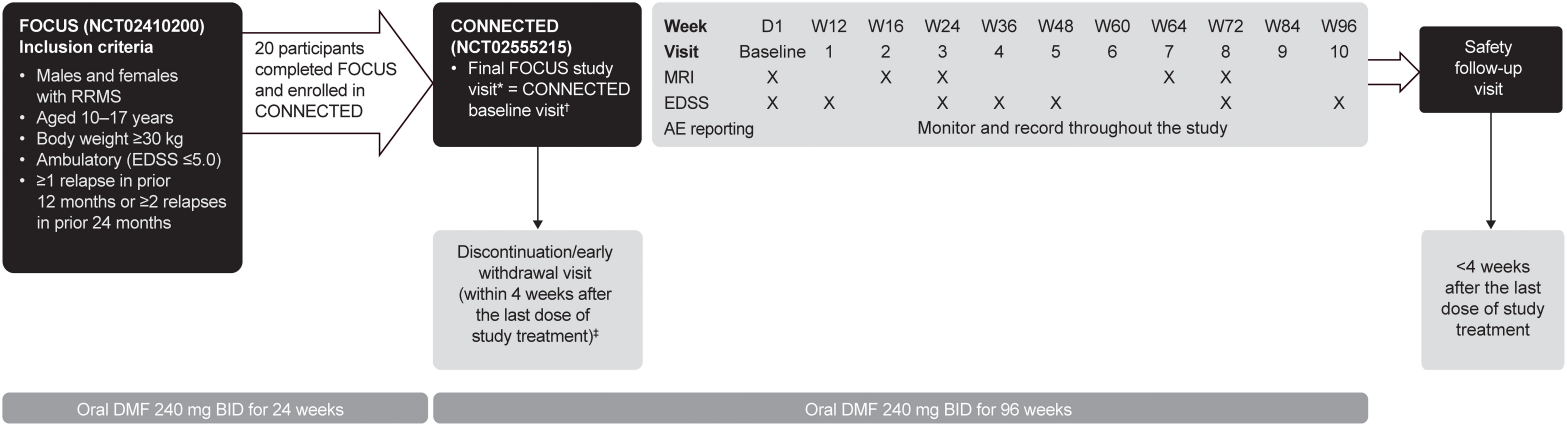
CONNECTED study design. AE, adverse event; BID, twice daily; D, day; DMF, delayed-release dimethyl fumarate; EDSS, Expanded Disability Status Scale; MRI, magnetic resonance imaging; RRMS, relapsing-remitting multiple sclerosis; W, week. ^*^Eligibility for CONNECTED was determined at the final study visit in FOCUS or within 4 weeks before CONNECTED study entry. ^†^Within 4 weeks of the final study visit for FOCUS, if the two visits could not have been held at the same time. ^‡^Participants who discontinued treatment early could remain in the study and continue protocol-required tests and assessments, and those who withdrew prematurely were encouraged to complete the safety follow-up visit.

CONNECTED is a 96-week (2-year) extension study of FOCUS, designed to evaluate the long-term safety, pharmacokinetics, and efficacy of DMF in pediatric patients with MS (31). During a 4-week enrollment period, eligible participants from the FOCUS study continued on DMF, underwent brain magnetic resonance imaging (MRI) scans, and were then enrolled into CONNECTED. At the end of the 4-week enrollment period, starting at day 1, participants received DMF 240 mg twice daily for 96 weeks (Figure 1). Participants then completed a safety follow-up visit up to 4 weeks after the last dose of study treatment. During the treatment period, clinic visits were conducted on day 1 and weeks 12, 16, 24, 36, 48, 60, 64, 72, 84, 96, and a safety follow-up visit. Follow-up brain MRIs occurred at weeks 16, 24, 64, and 72.

Key eligibility criteria included written informed consent from participants and their parent/legal guardian, completion of the FOCUS study per protocol, and continuing to receive DMF 240 mg twice daily. All female participants of childbearing potential and all male participants were advised to practice effective contraception during the study and for ≥30 days after their last dose of study treatment. The eligibility criteria for the FOCUS study included patients age 10–17 years at the time of enrollment, body weight ≥30 kg, and a diagnosis of RRMS according to both the McDonald (32) and the International Pediatric Multiple Sclerosis Study Group criteria for pediatric MS (33). Patients were also required to be ambulatory, with an Expanded Disability Status Scale (EDSS) score of ≤ 5.0, and had to have experienced at least one relapse in the 12 months or two relapses in the 24 months before screening. The main exclusion criteria were progressive MS, disorders mimicking MS, or a history of clinically significant comorbid disorders or conditions. Patients were also excluded if they received prior medications such as DMF (at any time); fingolimod, teriflunomide, or natalizumab (within 6 months before week −8 MRI); or glatiramer acetate, IFN-β, or corticosteroids (within 28 days before week −8 MRI).

For the CONNECTED study, the main exclusion criteria were unwillingness or inability to comply with the study requirements; any significant changes in medical history that occurred after enrollment; participants from FOCUS who could not have tolerated study treatment; a history of malignancy or severe allergic or anaphylactic reactions or known drug hypersensitivity to DMF or fumaric acid esters; abnormal blood tests [alanine aminotransferase >3 times the upper limit of normal (ULN), aspartate aminotransferase >3 times the ULN, gamma-glutamyl transferase >3 times the ULN, creatine >1.2 times the ULN, white blood cell count <2.0 × 10^9^/L, lymphocyte count <0.5 × 10^9^/L]; and female participants who were considering becoming pregnant.

### Study Endpoints

The primary endpoints were incidence rate and type of serious adverse events (SAEs), number and type of adverse events (AEs), and discontinuations of DMF due to an AE. Secondary endpoints included incidence of T2 hyperintense lesions from brain MRI scans over time, annualized relapse rate (ARR), EDSS scores over time, and the proportion of participants with confirmed disability progression.

### Analysis Populations

The safety population was defined as all participants who received at least one dose of DMF in CONNECTED. Analyses of safety data, including AEs, discontinuations due to AEs, clinical laboratory results, and vital signs were based on the safety population. The number of participants who were eligible for the study was determined by the number of participants who had completed FOCUS.

### Assessments

Safety assessments included AEs, vital signs, clinical laboratory parameters (chemistry, hematology, vitamin D levels, and urinalysis), and electrocardiograms. Efficacy assessments included ARR, MS relapse, EDSS, and MRI. MRIs were read at local sites. Participants had a protocol-defined relapse if they had new or recurrent neurologic symptoms lasting ≥24 h, not associated with fever or infection, accompanied by new objective neurological findings upon examination by the investigator. New or recurrent neurologic symptoms that occurred within 30 days of the onset of a protocol-defined relapse were considered part of the same relapse and were not treated with intravenous methylprednisolone per the protocol. Protocol-approved treatment for relapse was 3 or 5 days of intravenous methylprednisolone. Disability progression was measured by a ≥1.0-point increase in EDSS score from a baseline score of ≥1.0 sustained for 24 weeks, or a ≥1.5-point increase in EDSS score from a baseline score of 0 sustained for 24 weeks. Compliance with dosing was monitored by capsule count and captured in the electronic case report form.

### Statistical Analysis

ARR was calculated as the total number of relapses that occurred during the previous 12 months and during the 120 weeks on treatment for participants in FOCUS that continued into CONNECTED, divided by the total number of person-years followed prior to the study and by the total number of person-years followed during the study, respectively. MRI efficacy was evaluated using the total number of new or newly enlarging T2 hyperintense lesions on brain MRI scans from week 16 to week 24 and from week 64 to week 72. Summary statistics were presented for the primary endpoint data, pharmacokinetic parameters, and incidence of AEs. The 90% Hodges-Lehmann confidence interval (CI) was determined for the median change in the number of new or newly enlarging T2 hyperintense lesions (primary endpoint), and within-patient comparisons were made using the Wilcoxon signed-rank test to calculate *p* values for determination of statistical significance. All summaries and descriptive statistical analyses were performed using SAS version 9.4 (SAS^®^ Institute Inc., Cary, NC).

### Approvals

The study was approved by the institutional review board/independent ethics committee at each site and conducted in accordance with relevant US federal regulations, the Declaration of Helsinki, and the International Council on Harmonisation Guideline for Good Clinical Practice. The study protocol and amendments were approved by the relevant institutional ethics committees, and written assent and consent forms were obtained from each participant and his or her parent or legal guardian.

## Results

### Study Participants

Of the 20 participants who completed the 24-week FOCUS study, all 20 study participants were enrolled into the CONNECTED extension study conducted at 12 sites in 10 countries. At baseline in CONNECTED, median (range) age was 17 (14–18) years and the majority of participants were female (65%) (Table 1). More participants who continued from FOCUS into CONNECTED were aged 17 years [*n* = 7 (35%)] and 18 years [*n* = 6 (30%)] than 14, 15, and 16 years [*n* = 2 (10%), *n* = 2 (10%), and *n* = 3 (15%)], respectively.

**Table 1:** Baseline demographics (CONNECTED and FOCUS) and disease characteristics.

**Characteristic**	**All participants (*N* = 20)**
**FOCUS baseline**	
Median (range) time since first MS symptoms, years	2 (1–9)
Median (range) time since MS diagnosis, years	1 (1–7)
Mean (SD) no. of relapses in prior year	1.5 (0.9)
Mean (SD) no. of relapses in prior 2 years	2.1 (1.1)
Any prior MS treatment, *n* (%)	11 (55)
**CONNECTED baseline**	
Mean (SD) age, years	17 (1.3)
Age, years, *n* (%)	
14	2 (10)
15	2 (10)
16	3 (15)
17	7 (35)
18	6 (30)^a^
Female, *n* (%)	13 (65)
Race, *n* (%)^a^	
Asian	1 (5)
Black or African American	0
White	5 (25)
Other	14 (70)
Mean (SD) weight, kg	66.6 (12.1)
EDSS score, mean (range)	1.0 (0–3.5)
Mean (SD) vitamin D, pmol/L	182 (59)

*EDSS, expanded disability status scale; MS, multiple sclerosis; SD, standard deviation*.

a *Race and ethnicity were not reported for the majority of participants (14) due to confidentiality regulations*.

Prior to enrolling in FOCUS, 11 CONNECTED participants (55%) had received MS therapy. Of these, the most common medications were IFN-β-1a [*n* = 7 (35%)], glatiramer acetate [*n* = 2 (10%)], and IFN-β-1b [*n* = 2 (10%)].

The median (range) time on study (FOCUS and CONNECTED combined) was 99.4 (31.9–117.7) weeks. Median (range) compliance, calculated as the total number of DMF doses received divided by number of days on treatment, was 99.6% (92–100%). Seventeen participants (85%) completed the CONNECTED extension study; three participants discontinued DMF treatment and withdrew from the study because of an investigator decision (*n* = 2) or participant decision (*n* = 1) following the detection of new lesions on MRI scans. The patients left the study after 222, 532, and 599 days in the CONNECTED study, respectively.

### Safety

In CONNECTED, of the 20 participants who received at least one dose of DMF and were included in the safety population, 18 (90%) experienced one or more AEs. Most AEs were classified as mild [*n* = 12 (60%)] or moderate [*n* = 5 (25%)] (Table 2). The two most frequently reported AEs (≥20% of participants) were flushing [*n* = 5 (25%)] and MS relapse [*n* = 4 (20%)]. Headache, abdominal pain, upper respiratory tract infection, viral upper respiratory tract infection, cough, and dysmenorrhea were experienced by three participants each. Gastrointestinal events occurred from the start of CONNECTED through week 84, while flushing events occurred from the start of CONNECTED through week 60.

**Table 2:** Safety summary for the CONNECTED study.

**Variable, *n* (%)**	**All participants (*N* = 20)**
**Summary of AEs**	
Any AE	18 (90)
Any moderate or severe AE	6 (30)
Any severe AE	1 (5)
Any AE related to DMF	8 (40)
Any SAE	2 (10)
Any SAE related to DMF	0
Discontinued treatment due to AE	0
Withdrew from study due to AE	0
**Most common AEs (incidence** **≥20%)**	
Flushing	5 (25)
MS relapse	4 (20)
**AEs of special interest**	
PML or lymphopenia	0
Pancreatitis	0
Renal dysfunction	0
Hepatic dysfunction	0
Skin reaction	0
Rash	0

*AE, adverse event; DMF, delayed-release dimethyl fumarate; MS, multiple sclerosis; PML, progressive multifocal leukoencephalopathy; SAE, serious adverse event*.

Three participants experienced four SAEs during the CONNECTED study. Two participants experienced SAEs with an onset during CONNECTED: one with MS relapse and one with abdominal pain that required hospitalization. A third participant had an SAE of MS relapse that started during the FOCUS study and was ongoing at enrollment in CONNECTED. No SAEs were considered related to DMF, and no participants discontinued DMF or withdrew because of an AE (Table 2). No deaths were reported.

**Figure 2:**
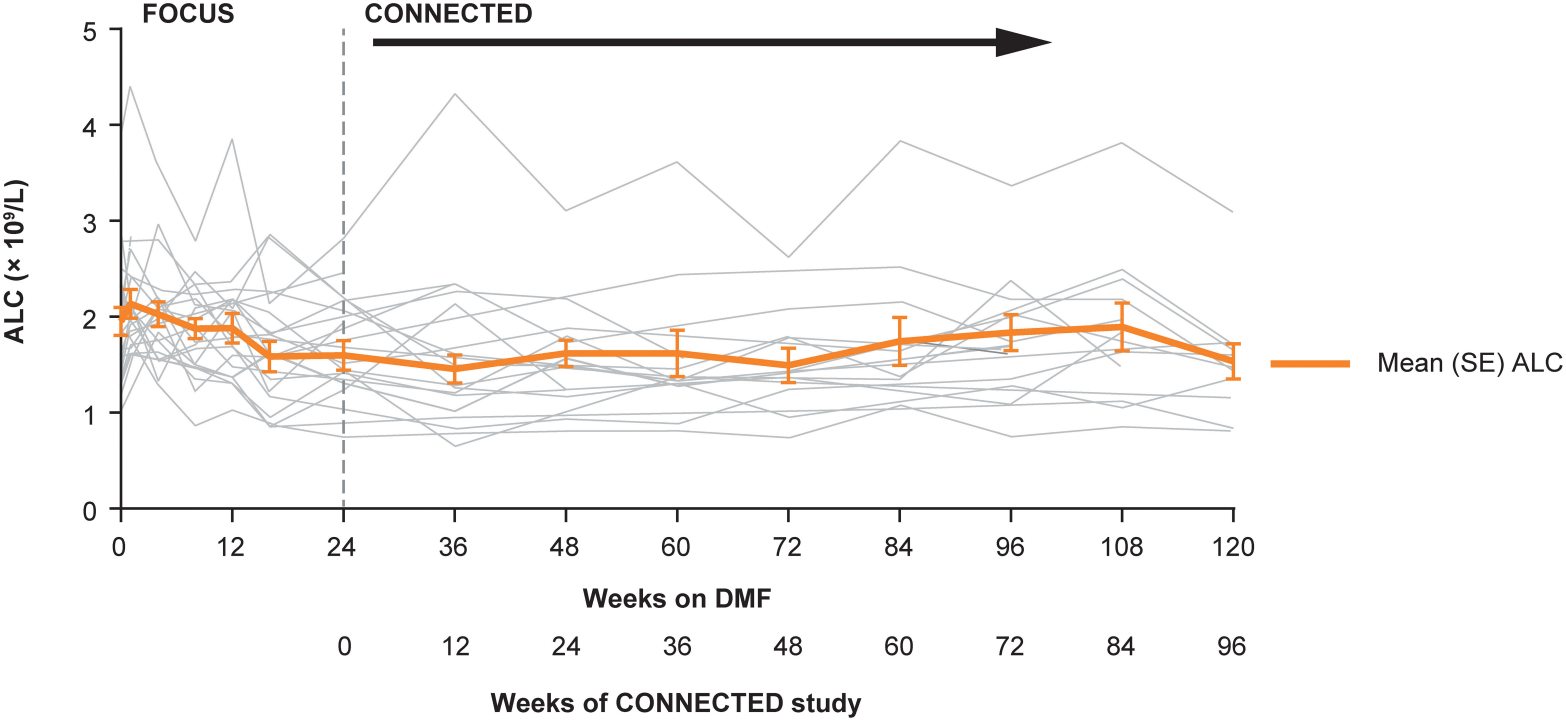
Individual and mean (SE) ALCs over time in the FOCUS and CONNECTED studies. ALC, absolute lymphocyte count; DMF, delayed-release dimethyl fumarate; SE, standard error.

Overall, mean absolute lymphocyte count in CONNECTED was 1.59 × 10^9^/L at baseline, 1.46 × 10^9^/L at week 12, and 1.53 × 10^9^/L at week 96 (Figure 2); the percentage change from CONNECTED baseline to week 96 was −3%. No participant had a lymphocyte count ≤ 0.5 × 10^9^/L and three participants had a lymphocyte count <0.8 × 10^9^/L during the study period. None of the low lymphocyte counts were associated with related AEs such as fever or infection. No participants experienced AEs related to pancreatitis, renal or hepatic dysfunction, or skin reaction or rash.

### Efficacy

In CONNECTED, of the 17 participants with an MRI evaluation at week 16 and week 24, 12 (71%) had no new or newly enlarged T2 lesions from week 16 to week 24, two (12%) had one lesion, and one participant (6%) had either two, three, or five or more lesions [median (range), 0 (0–6)]. Among 10 evaluable participants with MRI scans between week 64 and week 72 of CONNECTED, eight had no new or newly enlarging T2 hyperintense lesions, one participant had one lesion, and one participant had two lesions [median (range), 0 (0–2)] (Table 3).

**Table 3:** Incidence of new or newly enlarged T2 hyperintense lesions in the CONNECTED study.

	**Evaluable participants with MRI evaluation for new or newly enlarged T2 lesions**	
	**Between weeks 16 and 24 (*n* = 17)**	**Between weeks 64 and 72 (*n* = 10)**
**Number of lesions**, ***n*** **(%)**		
0	12 (71)	8 (80)
1	2 (12)	1 (10)
2	1 (6)	1 (10)
3	1 (6)	0
4	0	0
≥5	1 (6)	0
Mean (SD)	0.8 (1.6)	0.3 (0.7)
Median (range)	0.0 (0–6)	0.0 (0–2)

*MRI, magnetic resonance imaging; SD, standard deviation*.

**Figure 3:**
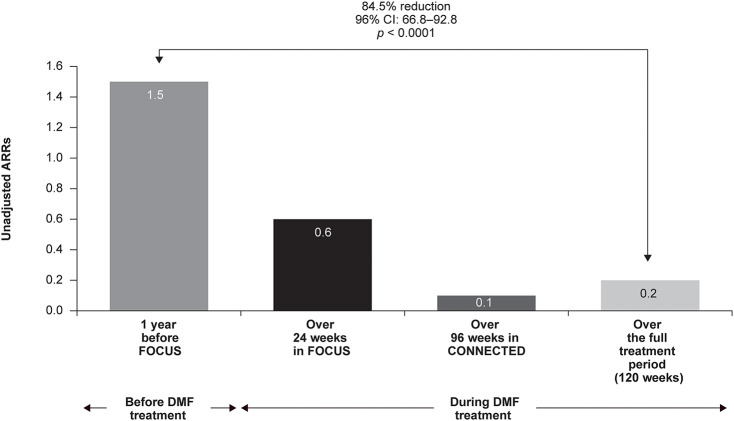
Annualized relapse rates (ARRs) in FOCUS and CONNECTED (*N* = 20). CI, confidence interval; DMF, delayed-release dimethyl fumarate.

Unadjusted ARRs were 1.5 for 1 year before FOCUS study entry, 0.6 after 24 weeks of DMF treatment, and 0.1 after 96 weeks of treatment in CONNECTED (Figure 3). Over the full 120-week treatment period encompassed by FOCUS and CONNECTED, ARR was 0.2, representing an 84.5% relative reduction in relapses (*n* = 20; 95% CI: 66.8–92.8; *p* < 0.0001) when compared with the year before treatment initiation.

At 1 year prior to enrollment into FOCUS, two of 20 participants had zero relapses, compared with 13 of 20 participants having zero relapses over 120 weeks on DMF treatment. The total number of relapses decreased from 29 relapses in 18 of 20 participants at 1 year prior to enrollment into FOCUS to six relapses in six of 20 participants during the 24-week period evaluated during FOCUS. The number of relapses further decreased to four in two of 20 participants during the 96 weeks evaluated during CONNECTED, for a total of 10 relapses in seven of 20 participants over 120 weeks on DMF treatment (Table 4). Two participants experienced a protocol-defined relapse requiring treatment with intravenous methylprednisolone; one of these participants was hospitalized due to the protocol-defined relapse.

**Table 4:** Relapses in the FOCUS and CONNECTED studies.

	**1 year prior to entry into FOCUS**	**FOCUS 24 weeks on DMF**	**CONNECTED 96 weeks on DMF**	**120 weeks on DMF**
	**(*n* = 20)**	**(*n* = 20)**	**(*n* = 20)**	**(*n* = 20)**
**Number of relapses**, ***n*** **(%)**				
0	2 (10)	14 (70)	18 (90)	13 (65)
1	9 (45)	6 (30)	0	5 (25)
2	8 (40)	0	2 (10)	1 (5)
3	0	0	0	1 (5)
4	1 (5)	0	0	0
≥5	0	0	0	0
**Total relapses**	**29**	**6**	**4**	**10**
**Total patient-years followed**	**20.0**	**9.5**	**35.0**	**44.5**

*DMF, delayed-release dimethyl fumarate. Bold values indicates that they are the total*.

In CONNECTED, no participant had an EDSS score ≥4 at baseline or at any time point evaluated (weeks 12, 24, 36, 48, 72, and 96); eight participants (40%) had baseline EDSS scores of 0. The median EDSS score was 1.0 at each time point evaluated, except for week 72 [median (range) EDSS score, 1.25 (0.0–3.5)]. The median change from baseline in EDSS score was 0 at each time point analyzed. Three participants met the protocol definition of disability progression during the study; two of these participants had an associated MS relapse, as described earlier. A third participant had a baseline EDSS score of 2.5 that increased to 3.5 from week 12 through week 72 and did not experience an AE of MS relapse associated with this increase in EDSS score. This participant had one new T2 lesion on MRI from week 16 to week 24 and no additional new or newly enlarged lesions from week 64 to week 72.

## Discussion

In this 96-week extension study of DMF treatment in pediatric patients with MS, DMF showed an acceptable long-term safety profile in pediatric and adolescent participants, consistent with that observed in adults. There were no unexpected observations regarding AEs, clinical laboratory parameters, vital signs, or electrocardiograms. No participants died during the study, discontinued DMF treatment due to an AE, or withdrew from the study due to an AE. Lymphocyte counts generally remained stable throughout study treatment (3% decrease from baseline to week 96), after an initial decline in FOCUS (31), and low lymphocyte counts were not associated with any related AEs such as infection or fever. In FOCUS, the mean percentage reduction in lymphocyte count from baseline to week 24 was 18% (31); in adult studies, the reduction was ~30% in the first 6 months−1 year (34).

This 96-week extension study confirmed and extended the findings from the 24-week FOCUS study, which demonstrated that DMF treatment is associated with a decrease in number of new or newly enlarging T2 hyperintense lesions [median (90% CI) change from baseline, −2.0 (−8.0, −1.5); *p* = 0.009] (31). Over 96 weeks, DMF treatment was also associated with a continued decrease in relapses and ARR. Similarly, in a retrospective chart review of patients ≤ 18 years of age with MS, ARR decreased and EDSS score was stable or decreased after ≥12 months of DMF treatment in all but one patient (*n* = 9) (30). In a randomized controlled trial of pediatric patients with MS, adjusted ARRs were 0.12 with fingolimod and 0.67 with IFN-β-1a (23). In CONNECTED, the unadjusted ARR was 0.1 for 96 weeks of DMF treatment and 0.2 over the full 120-week treatment period. In adults treated with DMF, ARR ranged from 0.08 to 0.22 (24, 35, 36) and 0.04 to 0.24 in newly diagnosed patients (36). Younger age of disease onset was associated with a lower relapse rate in patients treated with DMF in a retrospective analysis of an adult population (35).

In FOCUS, the most common AEs were gastrointestinal issues and flushing (31), as previously noted in other studies of DMF treatment in both pediatric (30) and adult participants with MS (24, 25). Overall, 91% of participants in FOCUS experienced an AE; six SAEs were reported in five participants, none related to DMF treatment (25). Gastrointestinal-related AEs were lower in CONNECTED than FOCUS (31), as would be expected because the majority of gastrointestinal-related AEs occur in adults in the first 5 weeks of DMF treatment (37).

Because this study is both small and single arm, the results should be interpreted with caution. Interpretation of results is also limited by the lack of race and ethnicity data due to confidentiality regulations for the majority of participants; no cognitive assessments collected in the study; and incomplete MRI outcomes for some patients. Although FOCUS was a pediatric study, the participants have aged over the CONNECTED extension study. These data represent adolescents, rather than children, as the majority of participants (13) were 17–18 years of age and only seven participants were 14–16 years of age. Given the nature of an extension study, as the participants aged, some of them moved into the older age group between the original FOCUS study and the current CONNECTED extension study. In addition, two participants in FOCUS did not roll over into CONNECTED and three participants discontinued during the CONNECTED study. The small sample size does not allow for a comparison of the younger and older participants.

Evidence suggests that pediatric-onset MS follows a clinical course that may be distinct from adult-onset MS; these differences may warrant specific treatment considerations (38). Pediatric patients may have more robust autoimmune responses compared with adult patients with MS (39–41), and central nervous system inflammation may have an impact on still-maturing immune and nervous systems (39). Hormonal changes, especially related to puberty, may affect MS susceptibility and the disease course (42). Pediatric patients tend to have a higher relapse frequency than adults, and more T2 lesions as shown on MRI (10, 11, 43), with more pronounced inflammation (44, 45). A good response to immunomodulatory treatments, including treatment with DMF, would be important for disease control. Patients with pediatric-onset MS were more likely to exhibit cognitive impairment and showed a faster decline in cognitive test scores than patients with adult-onset MS (46). Cognitive subdomains such as worse visual memory, as measured by 10/36 Spatial Recall Test, and information processing speed and executive functions, as measured by Symbol Digit Modalities Test, have been associated with relapses and possibly predictive of increased motor disability in pediatric and young (<25 years of age) patients with MS (47). Treating pediatric patients with effective DMTs early in the disease appears to reduce disease progression and may protect against or slow cognitive decline (48, 49).

Several treatment options are available to adult patients with MS; however, the treatment of pediatric patients with MS is currently limited owing to the lack of 2-year or longer data on the safety and effectiveness of the majority of MS drugs in pediatric patients. Evidence is emerging that it is important to treat children early, given the potential for cognitive difficulties and the earlier onset of disability progression. The “ideal” DMF patient has been suggested to be young and with short disease duration (35). This small single-arm study suggests that pediatric and adolescent patients with MS might benefit from treatment with DMF. Further studies are needed and are currently underway (ClinicalTrials.gov NCT02283853) to further inform clinicians on the effectiveness, safety, and suitability of DMF for treating pediatric patients.

## Data Availability Statement

All data generated or analyzed during this analysis are included in this published article/as supplementary information files.

## Ethics Statement

The studies involving human participants were conducted in accordance with relevant US federal regulations, the Declaration of Helsinki, and the International Council on Harmonisation Guideline for Good Clinical Practice. The study protocol and amendments were approved by: the University Medical Center in Göttingen, Germany; Commissie voor Medische Ethiek Universitair Ziekenhuis Gent, Gent, Belgium; LEC “MHATNP “Sv.Naum” EAD Sofia, Bulgaria; Ethics Committee Fakultni nemocnice Hradec Kralove, Hradec Kralove, Czechia; Research Affairs/Ethical Review Committee, Dasman Diabetes Institute “DDI” Dasman, Kuwait; Ethics Committee for Clinical Trials of Medicinal Products Riga, Latvia; American University of Beirut Institutional Review Board (AUB IRB) Beirut, Lebanon; the Independent Bioethics Committee for Scientific Research at Medical University of Gdańsk, Gdańsk, Poland; Hacettepe Universitesi Klinik Arastirmalar Etik Kurulu Ankara, Turkey; Loma Linda University Adventist Health Sciences Center, Institutional Review Board, Loma Linda, CA, United States. Written informed consent to participate in this study was provided by the participants' legal guardian/next of kin.

## Author Contributions

RA and PH designed and conceptualized the study, drafted the manuscript for intellectual content, had a major role in the acquisition of data, interpreted the data, and revised the manuscript for intellectual content. MM-B, AB, MV, and GA had a major role in the acquisition of data, interpreted the data, and revised the manuscript for intellectual content. JP analyzed and interpreted the data and revised the manuscript for intellectual content. XP interpreted the data and revised the manuscript for intellectual content. VB designed and conceptualized the study, drafted the manuscript for intellectual content, had a major role in the acquisition of data, interpreted the data, and revised the manuscript for intellectual content. All authors contributed to the article and approved the submitted version.

## Conflict of Interest

RA reports receiving personal compensation for serving on speaker/advisory boards for Bayer, Biogen, Merck, Novartis, Roche, and Sanofi, and receiving research support from Biogen, Merck, Novartis, and Roche for the establishment of regional multiple sclerosis registries and the conduct of clinical trials. PH reports receiving speaker/consulting fees from Bayer, Merck, and Novartis. AB reports receiving speaker/consulting fees from AveXis, Biogen, Genzyme, and Merck. MV reports receiving speaker/consulting fees from Biogen, Genzyme, Merck, Novartis, Roche, and Teva. JP is a contractor for Biogen. XP and VB are employees of and hold stock/stock options in Biogen. The remaining authors declare that the research was conducted in the absence of any commercial or financial relationships that could be construed as a potential conflict of interest.

## Abbreviations

AE: adverse event
ALC: absolute lymphocyte count
ARR: annualized relapse rate
BID: twice daily
CI: confidence interval
D: day
DMF: delayed-release dimethyl fumarate
DMT: disease-modifying therapy
EDSS: Expanded Disability Status Scale
IFN-β: interferon-beta
MRI: magnetic resonance imaging
MS: multiple sclerosis
PML: progressive multifocal leukoencephalopathy
RRMS: relapsing-remitting multiple sclerosis
SAE: serious adverse event
SD: standard deviation
SE: standard error
ULN: upper limit of normal
W: week.
