# Supplementary material for: Delayed-Release Dimethyl Fumarate Safety and Efficacy in Pediatric Patients With Relapsing-Remitting Multiple Sclerosis
Source: Front Neurol. 2021 Jan 4;11:606418. doi: 10.3389/fneur.2020.606418 (PMC7812971; doi:10.3389/fneur.2020.606418)
Supplement: Supplementary file 1 [file Table_1.docx]

**SUPPLEMENTARY TABLE 1 |** Treatment-emergent adverse events by Preferred Term in the CONNECTED study.

| **Adverse event by System Organ Class and Preferred Term, *n* (%)** | **All participants  (*N* = 20)** |
| --- | --- |
| **Patients with an event** | **18 (90)** |
| *Nervous system disorders* | 9 (45) |
| MS relapse | 4 (20) |
| Headache | 3 (15) |
| Hypoaesthesia | 2 (10) |
| Demyelination | 1 (5) |
| Migraine | 1 (5) |
| Paraesthesia | 1 (5) |
| Presyncope | 1 (5) |
| Syncope | 1 (5) |
| *Gastrointestinal disorders* | 8 (40) |
| Abdominal pain | 3 (15) |
| Abdominal pain upper | 2 (10) |
| Abdominal discomfort | 1 (5) |
| Diarrhoea | 1 (5) |
| Dry mouth | 1 (5) |
| Mouth ulceration | 1 (5) |
| Nausea | 1 (5) |
| Vomiting | 1 (5) |
| *Infections and infestations* | 8 (40) |
| Upper respiratory tract infections | 3 (15) |
| Viral upper respiratory tract infection | 3 (15) |
| Gastroenteritis | 2 (10) |
| Nasopharyngitis | 2 (10) |
| Pharyngitis | 2 (10) |
| Cystitis | 1 (5) |
| Herpes zoster | 1 (5) |
| Mastoiditis | 1 (5) |
| Respiratory tract infection viral | 1 (5) |
| Root canal infection | 1 (5) |
| Salpingo-oophoritis | 1 (5) |
| Sinusitis | 1 (5) |
| Tonsillitis | 1 (5) |
| *Respiratory, thoracic, and mediastinal disorders* | 5 (25) |
| Cough | 3 (15) |
| Oropharyngeal pain | 2 (10) |
| Rhinorrhoea | 2 (10) |
| Hyperventilation | 1 (5) |
| Paranasal cyst | 1 (5) |
| Productive cough | 1 (5) |
| Upper-airway cough syndrome | 1 (5) |
| *Vascular disorders* | 5 (25) |
| Flushing | 5 (25) |
| *Musculoskeletal and connective tissue disorders* | 4 (20) |
| Muscle spasms | 2 (10) |
| Arthralgia | 1 (5) |
| Back pain | 1 (5) |
| Limb discomfort | 1 (5) |
| Musculoskeletal pain | 1 (5) |
| *Reproductive system and breast disorders* | 4 (20) |
| Dysmenorrhoea | 3 (15) |
| Menstruation irregular | 1 (5) |
| Ovarian cyst | 1 (5) |
| Uterine inflammation | 1 (5) |
| *Skin and subcutaneous tissue disorders* | 4 (20) |
| Acne | 2 (10) |
| Alopecia | 1 (5) |
| Alopecia areata | 1 (5) |
| Dermatitis allergic | 1 (5) |
| Dry skin | 1 (5) |
| *General disorders and administration site conditions* | 3 (15) |
| Fatigue | 2 (10) |
| Influenza-like illness | 1 (5) |
| *Injury, poisoning, and procedural complications* | 3 (15) |
| Burns, second degree | 1 (5) |
| Fall | 1 (5) |
| Ligament sprain | 1 (5) |
| Limb injury | 1 (5) |
| *Ear and labyrinth disorders* | 2 (10) |
| Vertigo | 2 (10) |
| *Eye disorders* | 2 (10) |
| Eye irritation | 1 (5) |
| Vision blurred | 1 (5) |
| *Renal and urinary disorders* | 2 (10) |
| Dysuria | 1 (5) |
| Hypertonic bladder | 1 (5) |
| Micturition urgency | 1 (5) |
| Urinary retention | 1 (5) |
| *Cardiac disorders* | 1 (5) |
| Wolff-Parkinson-White syndrome | 1 (5) |
| *Immune system disorders* | 1 (5) |
| Dust allergy | 1 (5) |
| Seasonal allergy | 1 (5) |
| Smoke sensitivity | 1 (5) |
| *Psychiatric disorders* | 1 (5) |
| Irritability | 1 (5) |
| Somatic symptom disorder | 1 (5) |

*MS, multiple sclerosis.*

*A patient was counted once within each System Organ Class/Preferred Term.*
